# Thrips dynamics in *Allium* crops: Unraveling the role of reproductive mode and weather variables in *Thrips tabaci* population development

**DOI:** 10.1371/journal.pone.0314019

**Published:** 2025-01-24

**Authors:** Bettina Porta, Ben Vosman, Pablo González Barrios, Richard G. F. Visser, Guillermo A. Galván, Olga E. Scholten

**Affiliations:** 1 Plant Breeding, Wageningen University and Research, Wageningen, The Netherlands; 2 Departamento de Biología Vegetal, Facultad de Agronomía, Universidad de la República, Montevideo, Uruguay; 3 Graduate School Experimental Plant Sciences, Wageningen University and Research, Wageningen, The Netherlands; 4 Departamento de Biometría, Estadística y Computación, Facultad de Agronomía, Universidad de la República, Montevideo, Uruguay; 5 Departamento de Producción Vegetal, Centro Regional Sur (CRS), Facultad de Agronomía, Universidad de la República, Progreso, Canelones, Uruguay; University of Florida Institute of Food and Agricultural Sciences, UNITED STATES OF AMERICA

## Abstract

*Thrips tabaci* is the main thrips species affecting onion and related species. It is a cryptic species complex comprising three phylogenetic groups characterized by different reproductive modes (thelytoky or arrhenotoky) and host plant specialization. *Thrips tabaci* populations vary widely in genetic diversity, raising questions about the factor(s) that drive this diversity. We investigated the genetic diversity, reproductive mode, and heteroplasmy frequency in *T*. *tabaci* populations from different *Allium* spp fields in six locations in the Netherlands in 2021 and at two locations from the North and South of Uruguay over three years to unravel how the diversity is structured in the populations and if changes can be associated with weather variables. The thrips populations from each location studied were characterized by sequencing 33 individuals per sampling using the cytochrome oxidase subunit I (COI) gene. The reproductive mode was determined using specific primers and a phylogenetic analysis. Daily data for the weather variables was obtained from agrometeorological experimental stations located in the Uruguayan sampled crop fields. The diversity, reproductive mode, and heteroplasmy of *T*. *tabaci* populations in onion crops exhibited significant year-to-year variation depending on the location. Changes in the relative frequency of each reproductive mode in populations are associated with weather variables: precipitation, temperature, number of frosts, and relative humidity. Heteroplasmy frequency was associated with the same weather variables except temperature. In Uruguay and the Netherlands, *T*. *tabaci* thelytokous haplotype H1 was the most common, showing different heteroplasmy levels within and between the populations. In the field populations, a high frequency of heteroplasmic H1 individuals was associated with low precipitation, while all H1 individuals were also associated with high temperature and high relative humidity. In Uruguay, heteroplasmy was associated with arrhenotokous *T*. *tabaci* haplotypes, which were highly common in the North, pointing at specific adaptations leading to a faster population build-up. All this information may be instrumental for designing more precise integrated pest management techniques in both conventional and organic production.

## Introduction

Thrips feeding may cause more than 50% yield loss in onion cultivation [1, 2]. The main species of thrips that affect onion and other *Allium* species worldwide is *Thrips tabaci* Lindeman (Thysanoptera: Thripidae), also known as onion thrips [3]. Next to feeding damage, *T*. *tabaci* can transmit Iris yellow spot virus (IYSV), which can devastate an entire onion crop in only a few weeks [4]. Other thrips species reported in *Allium* spp. are *Frankliniella occidentalis* [3, 5], *Frankliniella fusca* [6], *Frankliniella intonsa* [3, 7], *Frankliniella tenuicornis* [3], *Thrips palmi* [3, 8] and *Scirtothrips dorsalis* [3, 9], of which the last two are reported in their centres of origin only [10, 11].

*Thrips tabaci* has its centre of origin in the Mediterranean region [12], which coincides with one of the onion’s secondary centres of origin [13]. However, nowadays, onion thrips is a major pest of agricultural crops worldwide [14]. Its polyphagous nature, high reproductive rate, short generation time, cryptic behaviour, relative ease to develop insecticide resistance, and ability to reproduce without mating (parthenogenesis) have led to this success [1, 12, 15]. Furthermore, *T*. *tabaci* populations show, within and among them, a remarkable variation [3] in both biological and ecological traits linked to the different phylogenetic groups [16].

*Thrips tabaci* populations may differ in host plant preference [17], the ability to transmit plant viruses [18], and insecticide resistance [19]. *Thrips tabaci* is a cryptic species composed of three genetic groups or lineages, determined by phylogenetic analysis based on the cytochrome oxidase subunit I (COI) mitochondrial DNA gene analysis. Each lineage is phenotypically characterized mainly by a specific reproductive mode and host association [16, 17].

The most common and worldwide distributed *T*. *tabaci* lineage is known as the ‘thelytokous leek group’ whose main hosts are leek and onion [3, 17, 20]. This lineage reproduces asexually by parthenogenesis and produces only female offspring [21, 22]. The second largest lineage is the ‘arrhenotokous leek group’ [3, 20], with the same main hosts [17]. They reproduce sexually, producing diploid females, and asexually via parthenogenesis, in which unfertilized eggs develop into haploid males [21, 22]. This lineage has a more localized distribution and is often found in sympatry with the thelytokous lineage [3]. Limited interbreeding between individuals from these two lineages has been reported [16, 22]. Interbreeding is also shown by the simultaneous occurrence of mitochondrial DNA haplotypes from these two phylogenetic groups into a single individual, called heteroplasmy [23], reported in both phylogenetic lineages [3]. The third phylogenetic lineage of *T*. *tabaci* is the ‘arrhenotokous tobacco group’, which was only reported in Eastern Europe and has tobacco as a host [16, 17].

*Thrips tabaci* populations may consist of different haplotypes in different frequencies, which may belong to different lineages. Populations without or with almost no genetic diversity were also found and composed of haplotype H1. Moreover, haplotype H1 showed a worldwide distribution [3]. Detailed knowledge of the genetic structure of *T*. *tabaci* populations and its evolution in cropping systems with onions will provide insights into the pest population ecology, which is necessary to develop effective management strategies [24].

Genetic characterization of thrips populations, using the sequence of the mtCOI gene at the individual level, makes it possible to determine the phylogenetic group present, the haplotype frequencies in the population, and the evolution of such diversity over time. Population genetics, along with environmental characterization, enables the generation of hypotheses on the ecological factors driving the population structure and phenotypic variation in traits related to thrips reproduction and host preference [16], as well as to obtain insights into the adaptative strategies used by the most successful haplotypes. The implications of heteroplasmy may be far-reaching, related to health and fitness and evolutionary population dynamics [25].

Heteroplasmy is reported in animals and is highly frequent in arthropods [23]. The phenomenon is commonly explained by the occurrence of paternal leakage [26–29]. In *T*. *tabaci* individuals, heteroplasmy was shown by the coexistence of mtCOI haplotypes of two different *T*. *tabaci* lineages [23]. The frequency of heteroplasmy in natural populations of arthropods can range from low, under 20% [30], to high in populations where all individuals are heteroplasmic [31]. In *T*. *tabaci*, heteroplasmy was reported in all thelytokous individuals analyzed from different populations collected across India [23]. Furthermore, in a recent study of *T*. *tabaci* populations on *Allium* hosts at 14 locations worldwide, heteroplasmy was detected in 66% and 13% of the arrhenotokous and thelytokous haplotypes, respectively [3]. Nevertheless, so far, no studies have been carried out on the dynamics of heteroplasmy in *T*. *tabaci* populations in time considering diversity (haplotype frequencies), reproductive mode, and genetic structure of the populations *in situ* and linking these to weather variables occurring. Such a study may shed light on the role of heteroplasmy in adaptation. We hypothesize that weather variables play a crucial role in heteroplasmy, reproductive mode, and genetic structure of thrips populations.

*Thrips tabaci* populations differ in the reproductive mode and genetic diversity [3], and seem to lack a correlation between the geographical and genetic distances [3, 32]. This might be the consequence of anthropogenic practices that lead to haplotype spread, followed by adaptation to the new environmental and climatic conditions. The genetic variation within the species might positively influence its adaptability under diverse climatic conditions [33, 34]. Genetic differentiation between *T*. *tabaci* populations from different Indian agroclimatic regions was reported. However, the research did not determine the reproductive mode and heteroplasmy in association with the weather variables occurring [33]. In another study, *T*. *tabaci* populations from the North and South of Uruguay showed differences in the haplotype composition and reproductive mode during one growing season [3], lacking information on heteroplasmy at the population level and the population dynamics over the years in association with the weather variables occurring.

In Uruguay, weather variables can differ sharply between years [35, 36] and between the Northern and Southern regions [36]. Onions are cultivated in both regions [37], which are around 500 km apart, and hardly in the in-between region. In both regions, *T*. *tabaci* has been a serious pest since 1952 [38, 39], causing direct and indirect damage [40–45]. In the Netherlands, *T*. *tabaci* is also a major pest in *Allium* spp. [46, 47]. Here *Allium* crop fields are distributed throughout the country, but extreme weather conditions between years are not as frequent [48] as in Uruguay [35, 36]. *Thrips tabaci* populations from *Allium* spp. fields at two Dutch locations showed the same reproductive mode and were genetically similar which was different from the situation in Uruguay [3], making a comparison between the two countries interesting.

This research aims to investigate if the genetic diversity, reproductive mode, and heteroplasmy frequency in *T*. *tabaci* populations changed in North and South Uruguay over three years and if the changes can be associated with weather variables, as well as in six locations in the Netherlands in 2021. We identified the most common haplotype in Uruguay and the Netherlands and the level of heteroplasmy in the populations to analyze whether heteroplasmy might confer adaptative advantages.

## Materials and methods

### Thrips sampling in the *Allium* fields in Uruguay and The Netherlands

In Uruguay, thrips were collected from onion fields for three years (2019, 2021, and 2022). In the South, during the first year, thrips were collected at the end of the growing season, while in the two following years, thrips were collected twice during the season, in the middle, and at the end of the growing season (S1 Table). In the North, where the onion cultivation period is shorter than in the South, we sampled in the late season of 2019 and during the late mid-season of 2021 and 2022 (S1 Table). We collected thrips adults and L1 larvae separately as repetition and as a potential estimator for the population composition at the end of the season (S1 Table). Thrips were collected from different plants spread throughout the field and separated by at least 1.5 m to avoid collecting individuals from the same offspring [23]. Thrips samples (n > 60) were stored in an Eppendorf tube containing 95% ethanol and kept at room temperature until DNA extraction. Thrips samples are referred to as location-time for the samples collected in the South of Uruguay and location-time-L1 or adult, representing L1 larvae or adults collected in the North of Uruguay.

In The Netherlands, onion or leek plants containing thrips were collected once at the end of the summer of 2021 at six locations throughout the country (Table 1). All locations were sampled for the first time, except De Kwakel, which was previously sampled early in the season of 2019 [3]. Thrips were kept alive and sent to Wageningen University and Research. To start a rearing, all the thrips and plant materials from one location were put inside a jar with a hollowed lid of 6 cm diameter and a 70-micron mesh to keep the thrips inside and alive for several days. After eight days, old leaves were removed, and the thrips were fed with thrips-free pieces of leek leaves every three to four days. After each rearing had stabilized (approximately 25 days ~ two *T*. *tabaci* generations later), a sample of more than 60 thrips was collected randomly and transferred into an Eppendorf tube containing 95% ethanol and stored at room temperature before DNA extraction. As thrips are invertebrate animals, approval from an ethics committee to manipulate them is not needed.

**Table 1:** *Thrips tabaci* Nei’s diversity index per sample collected in the North and South of Uruguay.

**Country**		**Location**	**Sampling date**	**Development stage of analyzed individual thrips**	**Nei’s diversity index**
Uruguay	North	Salto Grande	17.12.2019	Adults	1.1E-02
			01.11.2021	Adults	2.9E-04
			01.11.2021	L1 larvae	0
			04.11.2022	Adults	1.0E-02
			04.11.2022	L1 larvae	1.3E-02
	South	Progreso	19.12.2019	Adults	1.2E-02
			08.11.2021	Adults	1.6E-03
			08.12.2021	Adults	1.6E-04
			27.10.2022	Adults	6.1E-03
			25.11.2022	Adults	7.4E-03

Each sample is defined by the location, sampling date, and material type (adults or L1 larvae). Thirty-three thrips individuals were analyzed per sample. If a sample was composed of several thrips species, only the *T*. *tabaci* individuals within the sample were used to calculate Nei’s diversity index.

### Weather variables in the North and South of Uruguay

The daily data for the weather variables: temperature (°C), precipitation (mm), occurrence of frosts, and daily relative humidity at the locations in the North (Salto Grande) and South (Progreso) were obtained from the Experimental Stations belonging to the University of the Republic of Uruguay (CRS) and from the Agricultural National Research Institute (INIA) at the specified locations. For this study, we used the definition of frost as: A frost is a meteorological event on specific days during winter with temperatures below zero.

The daily data were used to calculate the average temperature, the accumulated rain, the average relative humidity per day, and the total number of days in which agrometeorological frosts occurred within the period from the onion transplanting date until the thrips sample collection date in 2019, 2021, and 2022 at each location.

### DNA extraction, COI amplification, sequencing, and species haplotype identification

Thirty-three individual thrips were randomly selected from each sample. DNA was extracted from each of the thirty-three thrips using the DNAeasy Blood & Tissue Kit (QIAGEN, Valencia, CA), according to the suppliers’ recommendations.

The COI gene fragment was amplified for each of the individual thrips using the universal COI primer pair, MTD7.2F ATTAGGAGCHCCHGAYATAGCATT and MTD9.2R CAGGCAAGATTAAAATATAAACTTCTG [49]. Twelve μl PCR solutions were prepared according to the manual of the Qiagen Kit (6 μl Multiplex PCR kit solution, 0.25 μl for each 10 nM primer MTD7.2F and MTD9.2R, 3,5 μl of thrips DNA and 2 μl ddH2O). The PCR amplification protocol involved a first 95˚C for 15 min step, then 35 cycles of 95˚C for 1 min, 50˚C for 1 min, and 72˚C for 1 min followed by a final extension at 72˚C for 10 min. The PCR products were sequenced forward and reverse using a 3500 ABI Sequencer (Applied Biosystems, USA). The consensus sequence was built using each band’s forward and reverse sequence in the package SeqMan Pro of the DNASTAR LASERGENE 17 program (DNASTAR, Inc., Madison, WI, USA). All the obtained consensus sequences were aligned in MEGA 7 [50] using CLUSALW [51]. Once sequences were aligned, forward and reverse primers were trimmed, and the resulting sequences were stored in a database. We used the Pop Art program [52] to identify all the haplotypes in the database and to construct a Median-joining haplotype network to visualize per haplotype the proportion of individuals per location as well as the mutational steps between the haplotypes detected in our study. To determine the thrips species to which the haplotype belonged, we conducted a BLAST analysis against the NCBI database. All the identified haplotypes were uploaded to the NCBI GenBank database under accession numbers PQ057455 to PQ057542.

### Reproductive mode determination of *T*. *tabaci* individuals

The *T*. *tabaci* reproductive mode was determined individually based on 1) to which *T*. *tabaci* phylogenetic group the COI haplotype belonged [3] and 2) the pattern of bands obtained in the reproductive mode-specific PCR reaction [53] for each individual *T*. *tabaci*. To perform the reproductive mode-specific PCR reaction a PCR amplification with the following three PCR primers was used: the universal primer TCOR-ATTGCGTAAATTATTCCTAAAAGTCCA plus the two strain-specific primers for each reproductive mode: 1) mtCOI-SSP TCOS-AACAGCTATTCTCCTTCTTTATCTC which amplifies a 261 nucleotides fragment in the case of arrhenotokous thrips and 2) mtCOI-SSP TCOC-GAACAGTATATCCACCTTTATCAACG which produces a 451 nucleotides fragment in the case of thelytokous thrips. If an individual thrips was heteroplasmic, both bands were amplified due to the coexistence of mtCOI haplotypes from different lineages [23, 54]. The PCR solution was prepared following the Qiagen kit manual with a total of 12 μl reaction mixture composed of 6 μl Multiplex PCR kit, 0.25 μl of each universal and strain-specific 10 μM primer, 0.5 μl of the 10 μM primer TCOR, 3.5 μl of thrips DNA and 1.5 μl ddH2O. The thermal cycling conditions started with an initial denaturation step at 95˚C for 15 min, followed by 35 cycles of 98˚C for 10 s, 60˚C for 1 min, and 68˚C for 1 min, and a final extension at 72˚C for 10 min. The PCR products were stained with RedSafe^™^ and separated on a 1.5% agarose gel, using the 100 bp DNA Ladder (Thermo Fisher Scientific) as reference.

### Intrapopulation haplotypes frequencies, heteroplasmy, and reproductive mode within *T*. *tabaci*

A database was created in Excel ^TM^ with 1) the identification number of the individual thrips, 2) the origin/location of the sample, 3) the thrips haplotype, 4) the *Thrips* species to which it belonged, 5) in the case of *T*. *tabaci* the band or bands produced after the reproductive mode specific PCR reaction; and 6) the 428-nucleotide sequence of the mtCOI gene fragment of the thrips. This database was first used to determine the proportion of *T*. *tabaci* per sample and the frequency (if present) of other thrips species haplotypes using MS Excel ^TM^. Secondly, considering only the *T*. *tabaci* individuals, we calculated and graphically displayed the *T*. *tabaci* haplotype frequency per sample and the frequency of the reproductive mode bands per haplotype using R Statistical Software (v4.1.2; R Core Team 2021). When only the 451-nucleotide band was present, the individual was considered thelytokous; when the 261-nucleotide band was present, the individual was considered arrhenotokous; and when both bands were present, the individual was considered heteroplasmic [3, 23]. In the case of heteroplasmic individuals, the reproductive mode was determined by the phylogenetic group to which the COI haplotype belonged [3]. For each location-time sample, we calculated the percentage of thelytokous and arrhenotokous individuals and, per haplotype, the percentage of heteroplasmic thrips if present.

Per sample, we depict the proportion of *T*. *tabaci* and the other thrips species (if present) and their haplotypes using a stacked bar chart. The haplotype composition of the *T*. *tabaci* populations at each location and sampling time and the proportion of heteroplasmic individuals per haplotype within the samples are presented in a specific combined chart generated in the R Statistical Software (v4.1.2; R Core Team 2021). The combined charts consist of a central pie chart that shows the different haplotypes frequencies within the *T*. *tabaci* sample, surrounded by a doughnut chart showing the frequency per *T*. *tabaci* haplotype of the stages (thelytokous, arrhenotokous, or heteroplasmic) detected by the reproductive mode specific PCR reaction.

### Nei’s genetic diversity within *T*. *tabaci* samples

Nei’s genetic diversity index of *T*. *tabaci* samples was estimated using MEGA 7 [50] as the number of base substitutions averaged over all pairwise comparisons among all the consensus sequences from the individuals within each sample. Nei’s genetic diversity index per sample represents the average evolutionary divergence across thrips sequence pairs among all the *T*. *tabaci* present in a specific sample.

### Phylogenetic analysis

A Neighbour-joining tree [55] was constructed for the 428 nucleotide mtCOI gene fragment, using the Mega Align Pro package version 17.1.1 (DNASTAR, Inc., Madison, WI, USA), to visualize the phylogenetic relationship among all sampled thrips haplotypes along with NCBI reference sequences for each *T*. *tabaci* reproductive mode and phylogenetic group, and to visualize the genetic distance among haplotypes and species. The tree was drawn to scale, with branch lengths representing the Nei’s genetic distances, estimated on the number of base substitutions per site, and computed using the Maximum Composite Likelihood method [56].

### Statistical analysis

To assess the variation in reproductive modes and heteroplasmy within Uruguayan and Dutch thrips populations, we employed the Chi-squared test and Pearson’s Chi-squared test with Yates’ continuity correction. These tests examined the differences in the frequencies of arrhenotokous and thelytokous individuals, as well as heteroplasmic and non-heteroplasmic individuals within the populations across northern and southern regions of Uruguay over several years and between the two regions as an aggregate.

Furthermore, for the Uruguayan populations, a binomial regression analysis was conducted to explore the relationship between reproductive mode and heteroplasmy frequency with the weather variables, temperature, precipitation, incidence of frosts, and average relative humidity. For the Dutch populations, the heteroplasmy frequencies were compared using logistic regression with a binomial distribution, considering location as a fixed effect. Post hoc comparisons of the estimated marginal means of heteroplasmy across locations were performed using Tukey’s test.

To visualize the distribution of heteroplasmic and non-heteroplasmic thrips among the reproductive modes per haplotype, a mosaic graph was constructed in R Statistical Software (v4.1.2; R Core Team 2021), encompassing all *T*. *tabaci* individuals from Uruguay. To determine if heteroplasmy distributes significantly differently among the haplotypes in the arrhenotokous and thelytokous reproductive modes, a Pearson’s Chi-squared test with Yates’ continuity correction was conducted.

Additionally, the Uruguayan data was compared to the heteroplasmy frequency in H1, the most prevalent haplotype, against the mean heteroplasmy across the other haplotypes sharing the same reproductive mode using a Chi-squared test. Binomial regression analysis was also applied to determine the effect of weather variables on the dominant haplotype and the presence of heteroplasmy within it, with significant effects identified at a 95% confidence level.

## Results

### Thrips species and haplotypes found in *Allium* spp. sampled in Uruguay and the Netherlands

**Fig 1:**
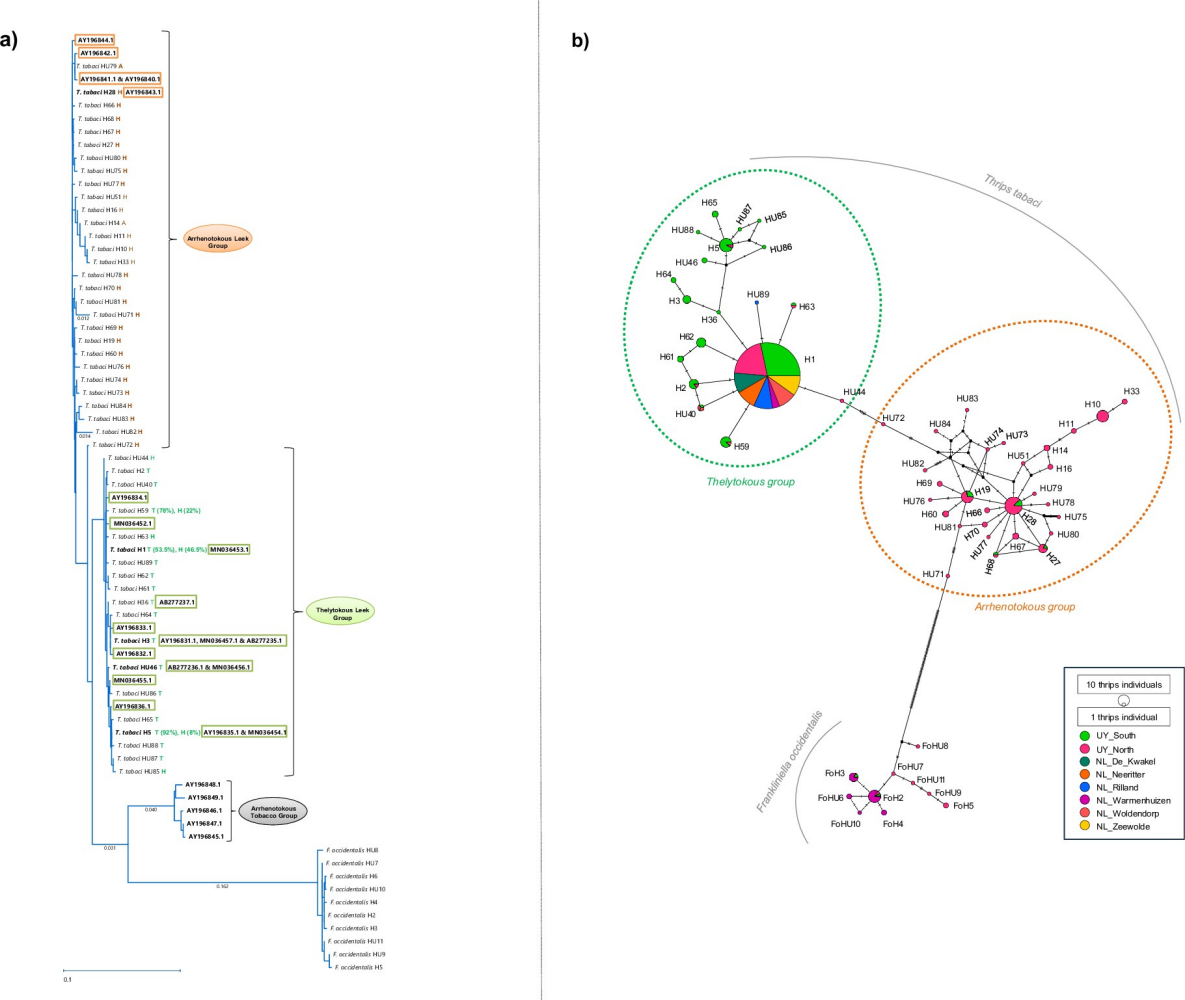
Phylogenetic relationship and Median-joining haplotype network of the thrips haplotypes. **a).** Phylogenetic relationship among the thrips haplotypes. Neighbour-Joining tree of the 58 *Thrips* COI haplotypes identified among all thrips samples from Uruguay (collected from 2019–2022) and the Netherlands (collected in 2021) and 25 *T*. *tabaci* NCBI accessions used as references for the reproductive modes and representing the different *T*. *tabaci* phylogenetic groups. The 58 COI haplotypes are represented by the abbreviation of the species to whom they belonged (*Thrips tabaci* or *Frankliniella occidentalis*) followed by H or HU and a specific number. The tree is drawn to scale, with the branch lengths representing the Nei’s genetic distance. Next to each *T*. *tabaci* haplotype, the result of the reproductive mode-specific PCR reaction is shown, where H represents heteroplasmy (both bands present in the individuals), A or T when only the Arrhenotokous or Thelytokous band was present, respectively. When a haplotype showed variable band patterns among the individuals, this is shown by the percentage of each type within brackets. The 25 NCBI accession numbers are in bold and within a rectangle. When a haplotype found in our study is synonymous with an NCBI accession, both are shown in bold. **b).** Median-joining haplotype network with the 58 thrips haplotypes found in Uruguay and the Netherlands. Haplotypes are represented by circles proportionally sized according to the number of thrips individuals. Different colours within the haplotypes represent proportionally the different origins of the individuals. Hatch marks indicate the mutational steps between haplotypes. The *Thrips tabaci* haplotypes that belonged to the thelytokous or arrhenotokous group are indicated by a dashed green and orange circle, respectively. *Frankliniella occidentalis* haplotypes are separated in the net by 85 mutation steps (hatch marks) from their closest *T*. *tabaci* haplotype HU71. UY and NL refer to Uruguay and The Netherlands, respectively, they are followed by the location where thrips populations were collected. The total number of thrips included in the net of haplotypes is 518.

A total of 518 thrips were analyzed using all samples collected in Uruguay and the Netherlands. These represented 58 different haplotypes, of which 48 belonged to *Thrips tabaci* and 10 to *Frankliniella occidentalis* (Fig 1). *Frankliniella occidentalis* was detected in the onion fields from the North and South of Uruguay in 2022, as well as in the Netherlands in Warmenhuizen in 2021 (S1 Fig and Fig 1B). Overall, it represented 6% of the individuals. *Thrips tabaci* was by far the most abundant species and represented 94% of all individuals analyzed. *Thrips tabaci* was present at all locations and sampling times and was the only species in most of the location-time samples (S1 Fig).

The *T*. *tabaci* haplotypes sampled from the onion and leek fields were identified as belonging to the arrhenotokous and thelytokous leek groups within the phylogenetic tree (Fig 1). Out of the total 48 *T*. *tabaci* haplotypes, nineteen were grouped in the thelytokous leek group, six of which had a few or more heteroplasmic individuals (Fig 1A). The remaining 29 haplotypes were part of the arrhenotokous leek group, of which 28 contained heteroplasmic individuals (Fig 1A).

Eighty-two percent of the *T*. *tabaci* individuals were classified as thelytokous based on the reproductive mode-specific PCR and the position of their haplotypes in the phylogenetic tree. These thrips originated from all locations and all sampling times. The remaining individuals were classified as arrhenotokous and originated mainly from the North of Uruguay.

Of the 48 *T*. *tabaci* haplotypes, haplotype H1 was the most frequent, representing 69% of the total *T*. *tabaci* and 84% of the thelytokous individuals analyzed (Fig 1B). Furthermore, *T*. *tabaci* haplotype H1 was the only one present at all locations (Fig 1B) and sampling times. Haplotype H1 frequency within samples varied widely, from one individual to all at certain locations and/or specific sampling times. The heteroplasmy level observed for haplotype H1 within the different samples also varied widely among the sampled locations and time points.

### *Thrips tabaci* diversity, reproductive mode, and heteroplasmy in Uruguay

**Fig 2:**
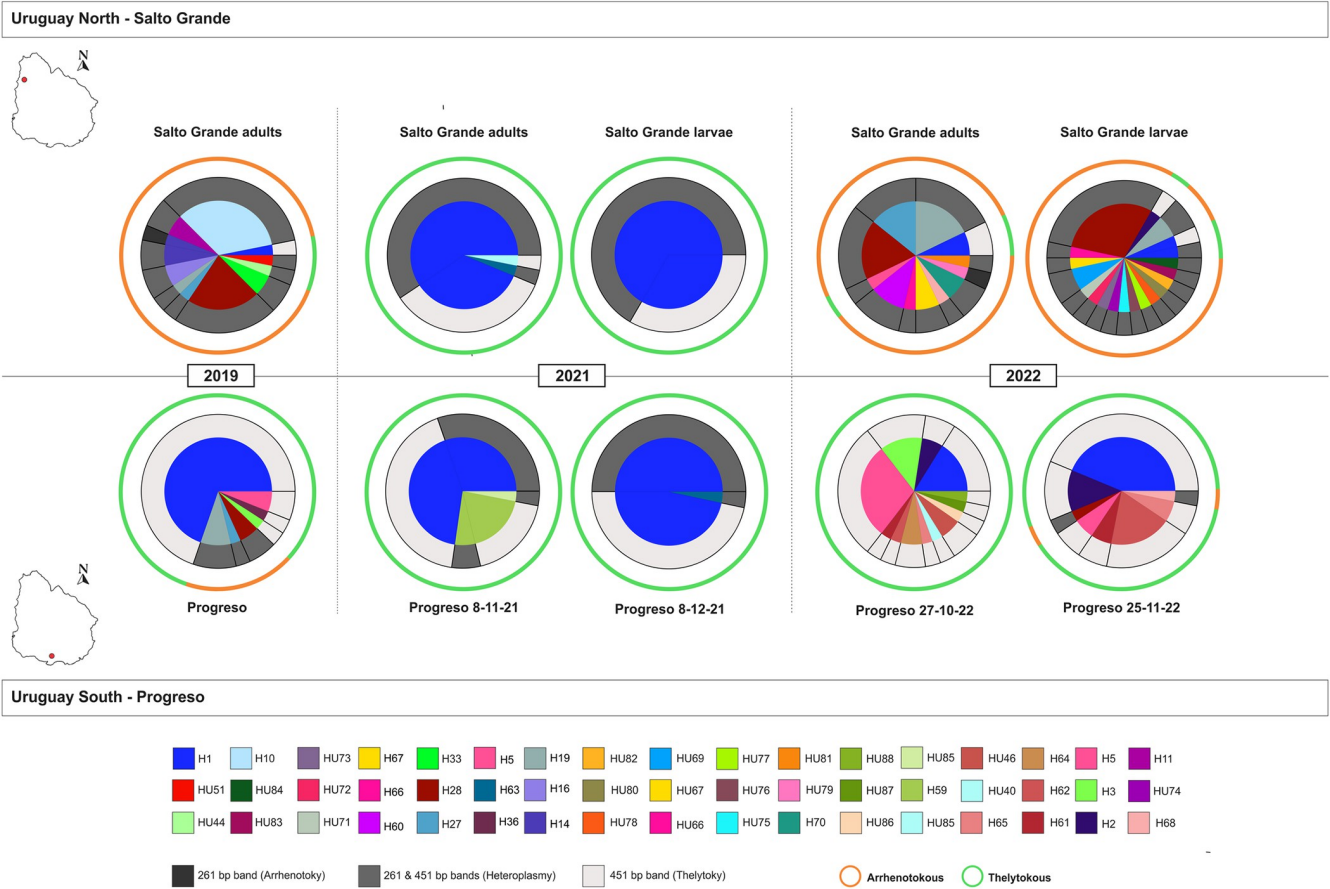
Haplotype frequency, heteroplasmy and reproductive mode in *T*. *tabaci* populations in Uruguay.

At both locations in Uruguay, the *T*. *tabaci* haplotype composition and frequencies per sample (Fig 2, S2 Table), along with Nei’s diversity index for each sample (Table 1), indicate that diversity was significantly higher in 2019 and 2022, compared to 2021. In 2021, diversity was zero in the L1 larvae sample from the North and close to zero in the three other samples collected in 2021 in the North and South (Table 1) due to the exclusive or near-exclusive presence of haplotype H1 (Fig 2, S2 Table).

Each graph represents a specific sampling during the 2019, 2021, and 2022 onion crop seasons at Salto Grande and Progreso (North and South of Uruguay, respectively). In each graph, the central pie chart shows the *T*. *tabaci* haplotype frequency within a sample, and each haplotype is represented by a different colour as indicated in the legend and named H or HU and a specific number. The doughnut graphs surrounding the pie charts show within each haplotype the percentage of individuals that showed only the arrhenotokous band (darkest grey tone), or only the thelytokous band (lightest grey tone), or the percentage of individuals that are heteroplasmic (middle grey tone), as determined by the reproductive mode specific PCR reaction. The external ring indicates to which phylogenetic group the haplotypes belonged, green for the thelytokous and orange for the arrhenotokous group.

In the North of Uruguay, haplotype H1 frequencies were notably low in 2019 and 2022, while in 2021, H1 became the predominant haplotype among adult thrips and was the sole haplotype found in L1 larvae (Fig 2, S2 Table). Conversely, in the South of Uruguay, haplotype H1 was dominant in 2019 and 2021. It was barely present in the sample collected in the middle of the 2022 season, but it regained dominance towards the end of that season (Fig 2, S2 Table).

#### Reproductive mode, heteroplasmy, and most common haplotype through the years in Uruguay

In 2021, *T*. *tabaci* populations from the North and South of Uruguay consisted exclusively of thelytokous individuals belonging mainly to haplotype H1 (Fig 2). In 2019 and 2022, a clear regional dichotomy was observed: in the North of Uruguay, 90% of the individuals were arrhenotokous, and 10% were thelytokous. In the same years, the opposite was observed in the South of Uruguay; 92% of the *T*. *tabaci* individuals were thelytokous, and 8% were arrhenotokous (Fig 2, S3 Table).

In 2019 and 2022, *T*. *tabaci* populations at both locations consisted of arrhenotokous and thelytokous individuals in sympatry. The frequencies of each reproductive mode per location-year showed significant differences (Table 2), as did the proportion of reproductive modes between years at each location. In 2021, only thelytokous individuals were present (Table 2). Also, the frequencies of each reproductive mode in the North and the South differed significantly (Table 2). Arrhenotokous individuals predominated in the North (except in 2021), whereas in the South the majority was thelytokous.

**Table 2:** Reproductive mode frequencies per year in the North and South Uruguay.

				**χ**^**2**^ **test for:**	**Pearson’s χ**^**2**^ **test with Yates’ continuity correction for:**	
**Location**	**Year**	**Rep. Mode**	**Frequency**	**the frequency of A and T per location-year**	**the frequency of A and T between years per location**	**the frequency of A and T in the North versus the South in the sympatric populations (2019 and 2022)**
				**p-value**	**p-value**	**p-value**
**North**	2019	A	0.94	7.43e-07 ***	2.88e-03 **	< 2.2e-16 ***
		T	0.06			
	2021	T	1.00			
	2022	A	0.90	1.54e-09 ***		
		T	0.10			
**South**	2019	A	0.18	2.57e-04***	3.48e-03 **	
		T	0.82			
	2021	T	1.00			
	2022	A	0.03	1.06e-13 ***		
		T	0.97			

The reproductive mode frequencies for A (arrhenotokous) and T (thelytokous) within each of the locations at a specific year and the χ^2^ test results are presented for the frequencies of A (arrhenotokous) and T (thelytokous) per site and year. Results of the Pearson χ^2^ test with Yates’ continuity correction for the frequencies of A and T between years per location and between the North and the South are shown in the 3^rd^ and 4^th^ main columns, respectively. Statistical significance when

p < 0.05 (*)

p < 0.01 (**), and

p < 0.001 (***).

The frequencies of heteroplasmic and non-heteroplasmic individuals differed significantly by location-year in all cases, except in the South in 2021 (S4 Table). In addition, the frequencies of heteroplasmic and non-heteroplasmic individuals showed significant differences between years within the North and the South. The frequencies of heteroplasmic and non-heteroplasmic thrips also varied significantly in the North and the South over the combined years (S4 Table), with heteroplasmy being more prevalent in the North than in the South (Fig 2).

In the North of Uruguay, in 2019 and 2022, 92% of the individuals were heteroplasmic, of which 95% belonged to arrhenotokous haplotypes and 5% to thelytokous haplotypes (Fig 2, S3 Table). In the South of Uruguay, all the heteroplasmic individuals in those years belonged to arrhenotokous haplotypes. In 2021, the percentage of heteroplasmy in the North and South of Uruguay was 65% and 46%, respectively, all in thelytokous individuals, most of whom belonged to haplotype H1 (Fig 2, S3 Table).

#### Weather variables associated with *Thrips tabaci* reproductive mode and heteroplasmy in Northern and Southern Uruguayan populations

Precipitation and temperature (S5 Table) were significantly associated with the proportion of arrhenotokous and thelytokous individuals (Table 3A). Arrhenotokous individuals were more common in conditions with higher precipitation and average to high temperatures. Meanwhile, thelytokous individuals were associated with lower precipitation. The number of frosts during the cropping season and the daily relative humidity (S5 Table) were significantly associated with the proportion of arrhenotokous and thelytokous individuals. Arrhenotokous individuals predominated in environments with a larger number of frosts and moderate to high relative humidity, while the thelytokous were prevalent in conditions with fewer frosts (Table 3B).

**Table 3:** Association between the weather variables in the North and South of Uruguay and *T*. *tabaci* reproductive mode.

**Reproductive mode in function of:**					
**a) Temperature and precipitation**					
**Coefficients:**					
** **	**Estimate**	**Std. Error**	**z value**	**Pr(>|z|)**	
**(Intercept)**	-16.96	3.74	-4.53	5.95E-06	***
**Temperature**	1.64	0.28	5.97	2.33E-09	***
**Precipitations**	-0.02	0.00	-9.36	< 2e-16	***
					
**b) Relative humidity and frosts**					
**Coefficients:**					
** **	**Estimate**	**Std. Error**	**z value**	**Pr(>|z|)**	
**(Intercept)**	24.72	7.17	3.45	5.64E-04	***
**Relative humidity**	-0.23	0.09	-2.60	9.30E-03	**
**Frosts**	-0.22	0.04	-6.30	2.90E-10	***

Binomial regression coefficients using the Uruguayan *T*. *tabaci* classified by its reproductive mode (arrhenotokous or thelytokous) as a factor in function of the weather variables: **a)** temperature (°C) and precipitation (mm); **b)** mean relative humidity per day (%) during the cropping period until sampling date and the number of frosts occurred in the same period.

Similar observations were made for heteroplasmy in the populations, except that temperature was not associated with heteroplasmy. We found that precipitation (S6A Table), the number of frosts, and the relative humidity (S6B Table) were significantly associated with heteroplasmy presence. In almost all cases, the heteroplasmic individuals were present under higher precipitation (S6A Table), except for the thelytokous heteroplasmic individuals that were mainly represented by H1. Higher relative humidity and number of frosts during the cropping season were linked to increased heteroplasmy in the populations (S6B Table).

#### Heteroplasmy per reproductive mode

Heteroplasmy was found in individuals from both arrhenotokous and thelytokous haplotypes (S2 Fig). Heteroplasmy was distributed significantly differently among the arrhenotokous and thelytokous haplotypes (p-value < 0.0001), being highly frequent within the arrhenotokous haplotypes (S2 Fig). Within the thelytokous haplotypes, the proportion of heteroplasmy was significantly different among the haplotypes and highest (41%) within haplotype H1 compared to the average for all other thelytokous haplotypes (11.5%) (p-value < 0.0001).

#### H1: Most common haplotype, heteroplasmy and its association with weather variables

Haplotype H1 emerged as the predominant haplotype in Uruguay. In the North, haplotype H1 frequency varied between years. In 2019, H1 represented 3% of the individuals, all non-heteroplasmic. An increase to 97% was observed in 2021, with 63% of these individuals being heteroplasmic. The proportion then dropped to 7% in 2022, showing a mere 2% heteroplasmy (Fig 2, S3 Table). In the South of Uruguay, haplotype H1 was the most common haplotype throughout the years (Fig 2, S2 and S3 Tables).

Lower precipitation, higher temperature, and relative humidity were significantly linked to increased frequencies of H1 in the populations whereas the number of frosts during the season was not (Table 4). A binomial regression analysis of heteroplasmy in H1 to weather variables showed that only precipitation was significantly associated with the probability of heteroplasmy in H1 (Table 4). For each millimetre increase in precipitation, the probability of occurrence of heteroplasmy is reduced by 1.4%.

**Table 4:** Binomial regression analysis.

**a) H1 in general**				
	**Estimate**	**Std. Error**	**z value**	**Pr(>|z|)**
**(Intercept)**	-60.718	11.281	-5.382	7.35e-08 ***
**Temperature**	2.266	0.364	6.223	4.87e-10 ***
**Precipitations**	-0.006	0.002	-2.656	7.9e-03 **
**Number of frosts**	0.008	0.026	0.331	0.7408
**Relative humidity**	0.387	0.097	3.998	6.39e-05 ***
**b) H1 heteroplasmic**				
	**Estimate**	**Std. Error**	**z value**	**Pr(>|z|)**
**Precipitations**	-0.014	0.006	-2.239	0.025 *

a) all Uruguayan haplotype H1 individuals and b) the Uruguayan heteroplasmic H1 individuals as a factor in function of the weather variables. In (b), only precipitation is reported, as it was the sole weather variable with a significant effect. Significance thresholds p < 0.05 (*), p < 0.01(**), and p < 0.001(***).

### Diversity, reproductive mode, and heteroplasmy of *Thrips tabaci* populations throughout the Netherlands in the late season of 2021

**Fig 3:**
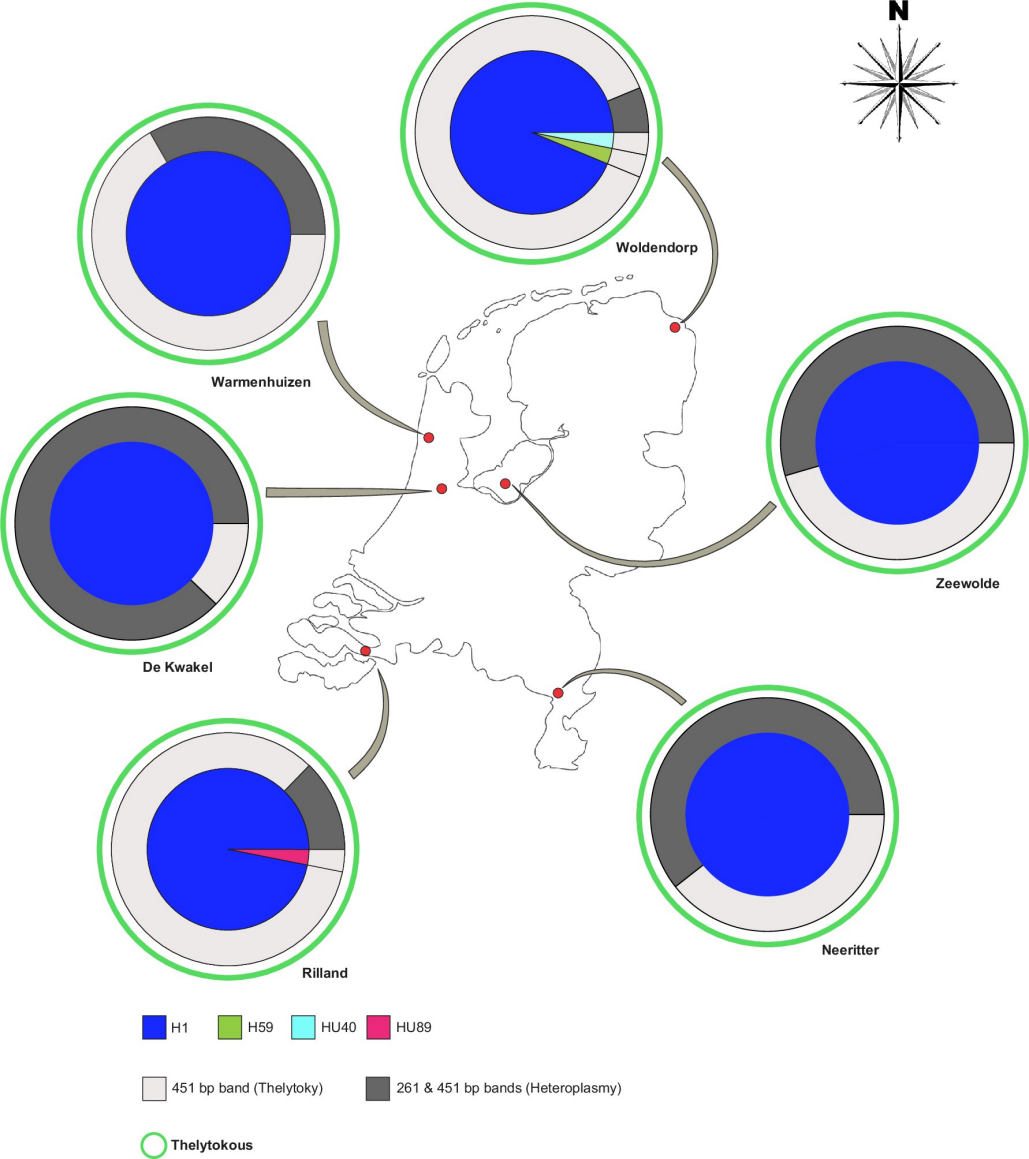
Haplotype composition, reproductive mode, and heteroplasmy of *Thrips tabaci* populations collected throughout the Netherlands. The haplotypes frequencies are depicted in the central pie chart, the percentages of heteroplasmy or presence of the thelytokous band in the individuals are shown in the surrounding doughnut chart in dark or light grey colours as shown in the reference, and the reproductive mode of the haplotypes based on their position in the phylogenetic tree is shown as a green external circle outside the pie and doughnut (duo) chart since all were thelytokous. Haplotypes are named H or HU plus a specific number and a colour to represent them.

The Dutch populations were not diverse in reproductive mode and displayed minimal diversity in haplotype constitution. However, they did show variability in heteroplasmy frequency. Four of the six samples collected across the Netherlands in 2021, De Kwakel, Neeritter, Warmenhuizen and Zeewolde, showed no genetic diversity and exclusively contained haplotype H1 (Fig 3). The exceptions were the samples from 1) Woldendorp, whose Nei’s diversity index was 2.9E-04, containing mainly haplotype H1 and one individual of each of the haplotypes HU40 and H59, and 2) Rilland, with a Nei’s diversity index of 1.5E-04. This population contained one individual of haplotype HU89, while all other individuals belonged to H1 (Fig 3, S2 Table).

All *T*. *tabaci* haplotypes identified in the Netherlands were classified as thelytokous based on their position in the phylogenetic tree and the presence of the thelytokous or both bands in the reproductive mode-specific PCR (Fig 3). Heteroplasmy levels varied significantly across the different locations (Fig 3). In De Kwakel, 88% of the individuals were heteroplasmic, followed by 61% in Neeritter and 55% in Zeewolde. The other three locations had fewer than 50% heteroplasmic individuals, Warmenhuizen (33%), Rilland (12%), and Woldendorp (6%). De Kwakel, Rilland, and Woldendorp populations showed significant differences in the frequencies of heteroplasmic and non-heteroplasmic individuals within samples based on the χ^2^ test under the hypothesis of equal distribution. When all Dutch populations were compared by their heteroplasmy frequency, they showed significant differences among them (Table 5).

**Table 5:** Heteroplasmy frequency within and between the Dutch *T*. *tabaci* populations.

			**a) χ**^**2**^ **test for:**	**b) Tukey test for:**
**Year**	**Location**	**Heteroplasmy frequency**	**Heteroplasmy proportions per location**	**Estimated marginal means heteroplasmy per location (groups)**
			**p-value**	
**2021**	**De Kwakel**	0.88	1.35e-05 ***	A
	**Neeritter**	0.61	0.22	AB
	**Rilland**	0.13	2.21e-05 ***	C
	**Warmenhuizen**	0.33	0.25	BC
	**Woldendorp**	0.06	7.43e-07 ***	C
	**Zeewolde**	0.55	0.6	AB

Frequency of heteroplasmic and non-heteroplasmic individuals in the Netherlands at the different locations in the late summer of 2021. Results of a) χ^2^ tests for heteroplasmy frequencies within location, significance thresholds p<0.05 (*), p<0.01(**) and p<0.001(***) and b) Tukey test for heteroplasmy estimated marginal means at each location, different letters between locations report significant differences (p<0.05) in the frequencies of heteroplasmy between locations.

## Discussion

The present research is the first study in onion that characterizes the dynamics in *T*. *tabaci* population diversity, heteroplasmy, and reproductive mode over three years at two locations and links these population data to changes in the main weather variables during and among growing seasons. We identified haplotype H1 as the most common haplotype in Uruguay and the Netherlands, which has different levels of heteroplasmy in populations, suggesting heteroplasmy as an adaptative advantage under specific conditions.

The genetic diversity in the Uruguayan *T*. *tabaci* populations is dynamic, changing during the crop’s growing season and over the years. This pattern aligns with the reported diversity trends for *T*. *tabaci* populations in New York in the mid and late seasons of 2005 and 2007 [57], as well as with the diversity observed in the Dutch population from De Kwakel sampled in the early season of 2019 [3] and in the mid-season of 2021 (present study). All cases showed more haplotypes in the early-season sampling and fewer haplotypes towards the end of the season, and generally, one of them turned dominant. Notably, the Uruguayan populations, particularly in the North, also exhibit changes in the reproductive mode of the haplotypes present over the years, alternating primarily between the arrhenotokous and thelytokous form, in contrast to the consistent thelytokous populations observed in De Kwakel and New York [3, 57]. Furthermore, the Uruguayan populations from the North and the South, which are geographically separated by approximately 500 km, differed in their frequencies of arrhenotokous and thelytokous individuals and the frequency of heteroplasmy.

We showed that in most cases, the frequency of heteroplasmic and non-heteroplasmic individuals within the populations is not equally distributed. At the same time, the heteroplasmy frequency differs between populations from different locations and years. Hence, heteroplasmy might be under genetic drift and/or natural selection [58, 59], and it might depend on the environmental conditions [60], like the local weather variables during the crop growing season. We hypothesize that the weather variables during the growing season play a role in determining the predominant reproductive mode and heteroplasmy frequency of the *T*. *tabaci* populations.

To find possible clues as to why the genetic composition of the Uruguayan populations changed so strongly in each growing season, we analyzed the association between the reproductive mode and heteroplasmy with weather variables in each season and location. We found that precipitation, temperature, daily relative humidity, and number of frosts are associated with the reproductive mode. The arrhenotokous haplotypes are successful under higher precipitation, temperature, relative humidity, and number of frosts, while the thelytokous are successful under low precipitation. The thelytokous were present under a wide range of temperatures, relative humidity, and number of frosts, which is consistent with their generalist nature as they are adapted to persist under a wide range of conditions [20]. Rain may drag down the larvae of the thrips [61, 62], which in combination with the longer development cycle and lower reproductive rate of the thelytokous in comparison to the arrhenotokous thrips [63], might turn the thelytokous less competitive when there is more precipitation.

The heteroplasmy frequency in these populations was associated with the same weather variables as the reproductive mode, except for the temperature, which was not associated with heteroplasmy. Heteroplasmy was mainly associated with the arrhenotokous haplotypes, but we cannot rule out that in certain Uruguayan environments, the heteroplasmic arrhenotokous *T*. *tabaci* might be positively selected for, leading to adaptation [64]. To shed light on this, experimental populations should be designed composed of arrhenotokous heteroplasmic and non-heteroplasmic individuals and analyze their dynamics under different sets of weather conditions. Heteroplasmy also occurred within the thelytokous haplotype H1. The frequency of the heteroplasmic H1 haplotype was significantly associated with lower precipitation, which might indicate a positive selection under dry weather and a negative one in case of high precipitation.

*Thrips tabaci* haplotype H1 was the most frequent and widely distributed haplotype in Uruguay and the Netherlands in the present, as well as in a former study on the diversity of thrips in *Allium* spp fields worldwide [3]. The high frequency and wide geographic distribution of certain haplotypes was also observed for H2 and H3 in *F*. *occidentalis* in the present and a former study [3]. All those haplotypes are examples of widely distributed invaders with selective advantages that make them highly competitive worldwide [65]. The high frequencies of *T*. *tabaci* haplotype H1 in the populations were associated with low precipitation, higher temperature, and relative humidity, which might indicate better competitive capacity under those conditions. Nevertheless, when these conditions are absent, H1 also remained in the populations at a low frequency, showing its wide adaptability and resilience, probably linked to the generalist nature of thelytokous [20]. It may be speculated that in a changing climate, with less rain and higher temperatures, H1 may become the dominant haplotype worldwide.

In the samples of the Netherlands that were derived from rearings, heteroplasmy was also observed, specifically associated with H1, with significant differences between populations. These differences might be traced back to the real situation in the fields since no significant changes are expected after around two generations [66]. However, we cannot rule out possible founder effects that might have occurred during the rearing [67]. Since we do not have specific data on the weather conditions in each field in the Netherlands, we could not explore its association with the weather conditions.

Our findings on the genetic structure of *T*. *tabaci* populations and the possible role of weather variables in shaping this warrants further research. Such research may include more locations and weather conditions, but also experimental work under controlled conditions with haplotype H1. An important issue is also how easily haplotype H1 develops resistance to insecticides, and if reproductive mode and heteroplasmy play a role in this. The development of insecticide resistance in *T*. *tabaci* populations has already been studied, but the populations involved were not characterized for reproductive mode [68]. Indirect damage caused by *T*. *tabaci* may also be linked to reproductive mode and possibly heteroplasmy. Variation in virus transmission between the two phylogenetic groups has already been shown [18]. It would also be interesting to study differential vector capacity for other pathogens like *Pantoea* spp [44]. All this information may provide clues for more precise integrated pest management techniques in both conventional and organic production [69].

## Conclusions

The diversity, reproductive mode, and heteroplasmy in *T*. *tabaci* populations on onion vary significantly between years and across locations. Populations with both phylogenetic groups (arrhenotokous and thelytokous leek groups, respectively) showed changes in their relative frequencies between years, being associated with precipitation, temperature, number of frosts, and relative humidity at the locations. The arrhenotokous thrips were highly frequent at high precipitation, temperature, relative humidity, and frosts during the season, while the thelytokous thrips dominate under lower precipitation. Via the dynamic changes both phylogenetic groups might be mutually cooperating to maintain their ecological niche and replace other *Thrips* species. Heteroplasmy was associated with precipitation, number of frosts, and relative humidity. It was independent of temperature. Heteroplasmy was also associated with the arrhenotokous haplotypes. Haplotype H1 was the most frequent haplotype in Uruguay and the Netherlands, showing variation in the heteroplasmy level within and between the populations, which might be linked to specific adaptations. In Uruguay, the H1 individuals were associated with lower precipitation, higher temperature, and relative humidity, while specifically, the heteroplasmic H1 individuals were associated with lower precipitations only. Further experiments are needed to determine which types of adaptations heteroplasmy might confer in haplotypes from the different phylogenetic groups. Furthermore, our findings on heteroplasmy in the arrhenotokous haplotypes and in H1 suggest that heteroplasmy might confer a higher reproductive rate under specific conditions, making the population build-up faster. A population composed of *T*. *tabaci* belonging to the arrhenotokous and thelytokous phylogenetic groups and with heteroplasmic individuals would be more hazardous to onion growers since such populations have better chances to build up quickly under various weather conditions.

## Supporting information

S1 Fig Frequency of *Thrips* species per sample at each location in Uruguay and the Netherlands. The (x) axis depicts the frequency. Each horizontal bar represents a different sample specified on the left. **A)** Depicts the frequencies of the *Thrips tabaci* (T.t species) and *Frankliniella occidentalis* (discriminated by haplotype composition) in the samples collected in the North and South of Uruguay, Salto Grande and Progreso, respectively, during 2019, 2021 and 2022. **B)** Species frequencies described as in A) for the samples collected in six locations distributed all over the Netherlands in 2021. The *Thrips tabaci* frequency per sample is represented as T.t species in blue. The haplotype composition in *T*. *tabaci* is not shown. The frequency of *Franklinella occidentalis* (F.o) is shown within a black rectangle by its stacked haplotype frequencies in the horizontal bars; a different colour represents each F.o haplotype. The different haplotypes are identified as F.o followed by an H or HU and a number.
(TIF)

S2 Fig Heteroplasmy frequency per reproductive mode and haplotype in Uruguay. On top is shown the proportions of arrhenotokous and thelytokous *T*. *tabaci* in Uruguay together with the frequency of heteroplasmy per reproductive mode. On the bottom is shown the frequency of heteroplasmy per haplotype in the Uruguayan samples. Arrhenotokous and thelytokous haplotypes are shown in bars grouped separately within the orange and green rectangle. The bar’s width is in scale with the proportion of each haplotype per reproductive mode in the Uruguayan samples. The proportion of heteroplasmic and non-heteroplasmic individuals per haplotype is represented in pink and sky blue, respectively. Heteroplasmy distribution among the haplotypes per reproductive mode (χ^2^ = 105.2, df = 1, p-value < 2.2e-16 ***). Heteroplasmy distribution within thelytokous; H1 (41%) against the mean of all other thelytokous haplotypes (11.5%) (χ^2^ = 16.3, df = 1, p-value = 5.4e-05 ***).
(TIF)

S1 Table Passport data of the thrips samples collected in Uruguay from 2019 until 2022 and in the Netherlands in 2021. (XLSX)

S2 Table Thrips species haplotype frequencies in the samples collected in Uruguay from 2019 until 2022 and in the Netherlands in the late season 2021. *Thrips tabaci* is abbreviated as T.t, and *Frankliniella occidentalis* is abbreviated as F.o. After the species abbreviation, the different haplotypes are named with H or HU followed by a specific number.
(XLSX)

S3 Table Individual thrips database. Each thrips analyzed has a specific identification number, sample origin (location and collecting date), thrips species identification, and haplotype identification. In the case of *T*. *tabaci*, the reproductive bands obtained after the reproductive mode specific PCR are reported. When the double bands 261 and 451 are observed, it means it is a heteroplasmic *T*. *tabaci* individual. The sequence of the mtCOI gene fragment (428 nucleotide length) is reported for all the thrips analyzed.
(XLSX)

S4 Table Frequency of heteroplasmic *T*. *tabaci* in Uruguay by location-year and statistical tests within and between populations and locations. The χ^2^ test was used to compare the frequencies per location-year (2^nd^ main column), and the Pearson χ^2^ test with Yates’ continuity correction was used to compare the frequencies found between years within locations (3^rd^ main column) and between the North and the South considering all the years (4^th^ main column). Statistical significance when p<0.05 (*), p<0.01(**) and p<0.001(***).
(XLSX)

S5 Table Weather variables at the sampled fields in the North and South of Uruguay. Mean temperature (°C), accumulated rain (mm), number of frosts and mean relative humidity (%) from the planting date until the collecting date of the thrips samples in the locations of Salto Grande and Progreso in the North and South of Uruguay respectively. Data is presented in conditional formatting with bars.
(XLSX)

S6 Table Association between the weather variables in the North and South of Uruguay and *T*. *tabaci* heteroplasmy presence. Binomial regression coefficients using the Uruguayan *T*. *tabaci* condition of heteroplasmic or non-heteroplasmic as a factor in function of the weather variables: **a)** temperature (°C) and precipitation (mm); **b)** mean relative humidity per day (%) during the cropping period until sampling date and the number of frosts occurred in the same period.
(XLSX)
