# Supplementary figures and images for: Thrips dynamics in Allium crops: Unraveling the role of reproductive mode and weather variables in Thrips tabaci population development
Source: PLoS One. 2025 Jan 24;20(1):e0314019. doi: 10.1371/journal.pone.0314019 (PMC11760593; doi:10.1371/journal.pone.0314019)

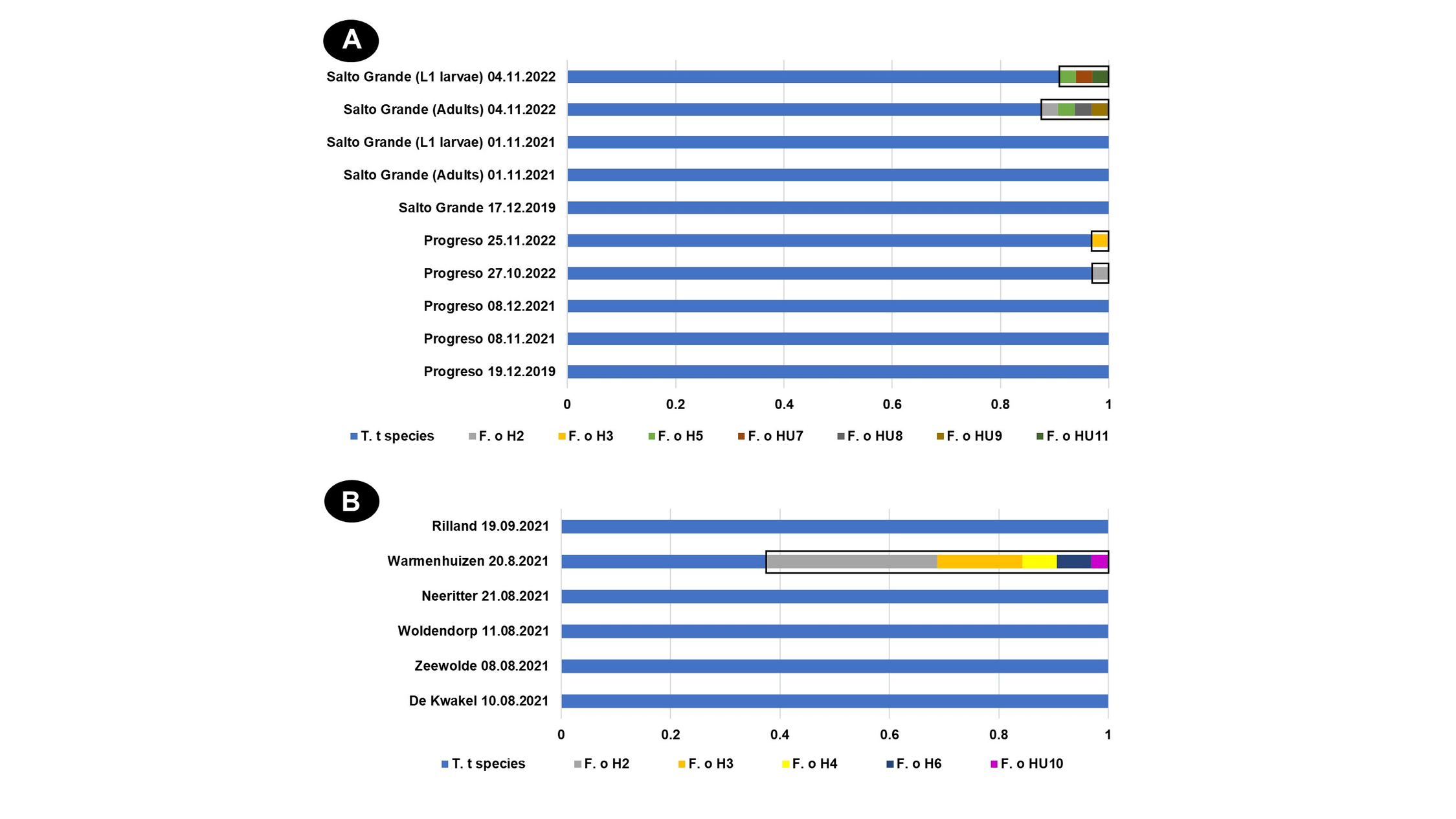

Supplement: S1 Fig — The (x) axis depicts the frequency. Each horizontal bar represents a different sample specified on the left. A) Depicts the frequencies of the Thrips tabaci (T.t species) and Frankliniella occidentalis (discriminated by haplotype composition) in the samples collected in the North and South of Uruguay, Salto Grande and Progreso, respectively, during 2019, 2021 and 2022. B) Species frequencies described as in A) for the samples collected in six locations distributed all over the Netherlands in 2021. The Thrips tabaci frequency per sample is represented as T.t species in blue. The haplotype composition in T. tabaci is not shown. The frequency of Franklinella occidentalis (F.o) is shown within a black rectangle by its stacked haplotype frequencies in the horizontal bars; a different colour represents each F.o haplotype. The different haplotypes are identified as F.o followed by an H or HU and a number. (TIF) [file pone.0314019.s001.tif]

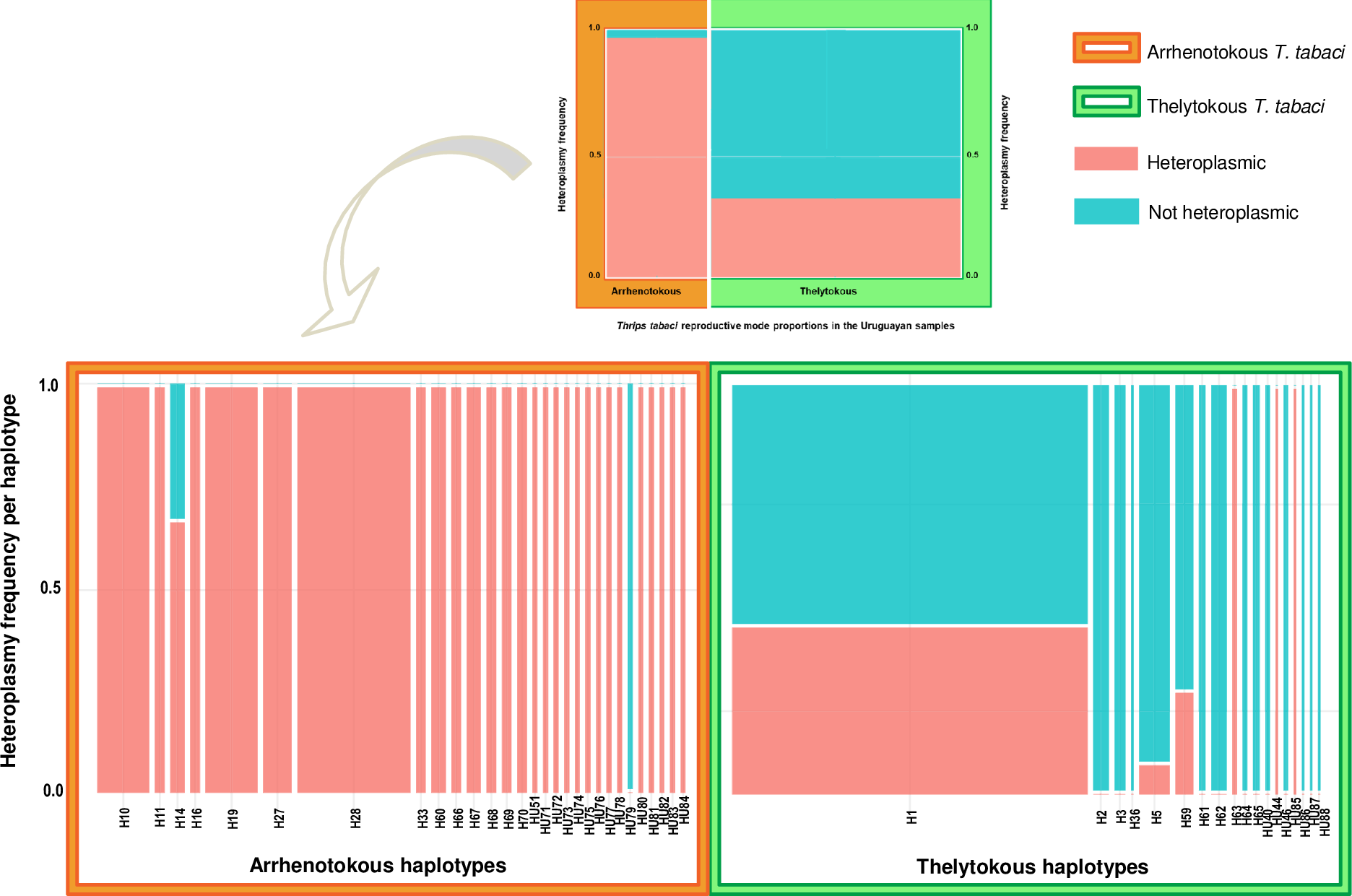

Supplement: S2 Fig — On top is shown the proportions of arrhenotokous and thelytokous T. tabaci in Uruguay together with the frequency of heteroplasmy per reproductive mode. On the bottom is shown the frequency of heteroplasmy per haplotype in the Uruguayan samples. Arrhenotokous and thelytokous haplotypes are shown in bars grouped separately within the orange and green rectangle. The bar’s width is in scale with the proportion of each haplotype per reproductive mode in the Uruguayan samples. The proportion of heteroplasmic and non-heteroplasmic individuals per haplotype is represented in pink and sky blue, respectively. Heteroplasmy distribution among the haplotypes per reproductive mode (χ2 = 105.2, df = 1, p-value < 2.2e-16 ***). Heteroplasmy distribution within thelytokous; H1 (41%) against the mean of all other thelytokous haplotypes (11.5%) (χ2 = 16.3, df = 1, p-value = 5.4e-05 ***). (TIF) [file pone.0314019.s002.tif]
